# Four-dimensional Plan Optimization for the Treatment of Lung Tumors Using Pencil-beam Scanning Proton Radiotherapy

**DOI:** 10.7759/cureus.3192

**Published:** 2018-08-23

**Authors:** David Cummings, Shikui Tang, William Ichter, Peng Wang, Jared D Sturgeon, Andrew K Lee, Chang Chang

**Affiliations:** 1 Physics, Texas Center for Proton Therapy, Irving, USA; 2 Medical Physics, Texas Center for Proton Therapy, Irving, USA; 3 Physician, Texas Center for Proton Therapy, Irving, USA; 4 Radiation Oncology, Texas Center for Proton Therapy, Irving, USA

**Keywords:** 4d robust optimization, robust optimization, proton pbs treatment, interplay effect

## Abstract

Purpose

This study aimed to evaluate the effectiveness of four-dimensional (4D) robust optimization for proton pencil-beam scanning (PBS) treatment of lung tumors.

Patients and methods

In seven patients with lung cancer, proton beam therapy was planned using 4D robust optimization over 4D computed tomography (CT) data sets. The gross target volume (GTV) was contoured based on individual breathing phases, and a 5-mm expansion was used to generate the clinical target volume (CTV) for each phase. The 4D optimization was conducted directly on the 4D CT data set. The robust optimization settings included a CT Hounsfield unit (HU) uncertainty of 4% and a setup uncertainty of 5 mm to obtain the CTV. Additional target dose objectives such as those for the internal target volume (ITV) as well as the organ-at-risk (OAR) dose requirements were placed on the average CT. For comparison, three-dimensional (3D) robust optimization was also performed on the average CT. An additional verification 4D CT was performed to verify plan robustness against inter-fractional variations.

Results

Target coverages were generally higher for 4D optimized plans. The difference was most pronounced for ITV V70Gy when evaluating individual breathing phases. The 4D optimized plans were shown to be able to maintain the ITV coverage at full prescription, while 3D optimized plans could not. More importantly, this difference in ITV V70Gy between the 4D and 3D optimized plans was also consistently observed when evaluating the verification 4D CT, indicating that the 4D optimized plans were more robust against inter-fractional variations. Less difference was seen between the 4D and 3D optimized plans in the lungs criteria: V5Gy and V20Gy.

Conclusion

The proton PBS treatment plans optimized directly on the 4D CT were shown to be more robust when compared to those optimized on a regular 3D CT. Robust 4D optimization can improve the target coverage for the proton PBS lung treatments.

## Introduction

Lung cancer is the second most common malignancy among both male and female populations in the U.S., and it is the leading cause of cancer-related mortality, accounting for one out of four cancer deaths in 2017 [1]. An estimated 222,500 new cases of lung cancer were diagnosed, and approximately 156,000 died of the disease in 2017. Proton radiotherapy, in the absence of an exit dose, can provide an escalated prescription target dose with limited toxic side effects [2-4]. The advent of the spot scanning proton delivery technique enabled the development of intensity-modulated proton therapy (IMPT) that can potentially further improve the effectiveness of proton radiotherapy for lung cancer [5-6]. However, for IMPT, the effect of breathing motion, i.e., the interplay between the moving lung anatomy and the sequentially delivered proton spots, needs to be mitigated in order to ensure accurate dose distribution [7-14]. Strategies such as four-dimensional (4D) robust optimization [15-16], gating [17], repainting [18], compression belt [19], synchronized multi-gating [20-23] and breath-hold [24] have been shown to provide superior dose distributions for proton lung treatments. Here, we examined the clinical use of 4D robust optimization in a commercially available treatment planning system (TPS) for pencil-beam scanning (PBS) treatment of lung tumors.

Conventionally, lung cancer treatments are planned based on average computed tomography (CT) scans. A 4D CT is performed by monitoring the patient’s breathing motion while scanning. The scan data are then binned into 8 to 10 breathing phases and reconstructed to obtain a 4D CT image set. An average CT image is generated by averaging all the breathing phases across the 4D CT image set. A maximum intensity projection (MIP) image is also generated by taking the highest Hounsfield unit (HU) encountered in each voxel throughout the breathing cycle [25-26]. By reconstructing different breathing phases, one can visualize tumor motion throughout the breathing cycle in the 4D CT. Note that tumor motion is also captured by the average CT and MIP images, although without time resolution; traditionally, treatment plans are designed and optimized based on the average CT scan alone.

In this study, we compared three-dimensional (3D) optimization plans that were optimized on the average CT with 4D optimization plans that, in addition to the average CT, were also optimized on the individual breathing phases of the 4D CT. Robust target and organ-at-risk (OAR) objectives were used for both 3D and 4D optimizations in this study. The same internal target volume (ITV) contour was used in both optimizations for each patient, while the gross target volume (GTV) and clinical target volume (CTV) contours were defined in the individual breathing phases. The effectiveness of 4D robust optimization was also evaluated against inter-fractional variations using a verification CT.

## Materials and methods

Seven patients previously treated for lung tumors were identified from the clinical database. All of these patients were simulated with 4D CT in the supine position. Breathing motion was monitored using the Anzai respiratory gating system with a laser sensor (AZ-733VI, ANZAI Medical, Japan).

An average CT image was generated for each 4D CT data set. For lung tumors, the HU value of the lesion is typically greater than that of the surrounding tissues, and consequentially, the extent of GTV motion can be easily delineated using MIP images. The internal gross tumor volume (IGTV) is thus defined on the MIP image to account for the GTV motion, and the ITV is typically obtained as an expansion of the IGTV. These contours were then transferred to the average CT image set for the conventional design and optimization of the proton treatment plan. As both the average CT and 4D CT have the same frame of reference, contour transfer between the CT images was done without additional image registration. Note that since the consistency between the ITV contours defined using the MIP images and those generated by combining individual CTV contours from all breathing phases may be variable [27-29], contouring procedures were performed in all patients in this study as outlined above.

**Figure 1:**
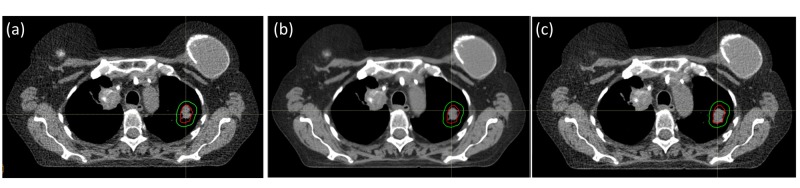
Target contour definition The ITV (green) and IGTV (red) contours as observed in the (a) maximal inspiration phase, (b) average CT scan and (c) maximal expiration phase of the breathing cycle. Note that the IGTV has been contoured directly on the MIP and transferred to the individual breathing phase CTs. ITV: internal target volume, IGTV: internal gross target volume, MIP: maximum intensity projection

For the purpose of this study, all treatment plans were normalized to a standard prescription dose of 70 Gy(RBE) in 35 fractions at 2 Gy(RBE) per fraction. Both 3D and 4D treatment plans for each patient were generated using the same planner to ensure consistency while comparing treatment plans. For 3D robust optimization, the HU value of the IGTV contoured on the average CT images is overridden to the average HU value of the IGTV defined using the MIP images. A uniform expansion of 5 mm was used to create the ITV from the IGTV. For 4D robust optimization, the GTV was contoured on individual breathing phase of 4D CTs and expanded with a 5-mm margin to obtain the CTV. An example of the target contours is shown in Figure 1. For 4D robust optimization, all the target objectives were placed on the GTV and CTV contours of the individual breathing phases. Besides, for both 3D and 4D robust optimization, the target objectives were placed on the ITV contours. The gantry and couch angles were kept the same for the 3D and 4D treatment plans for each patient, i.e., the only difference between the 3D and 4D treatment plans was the inclusion of the individual breathing phases for optimization.

The OARs include the lungs, esophagus, heart, spinal cord and, in some cases, the liver. All OARs were contoured directly on the average CT images. If the tumor was close to or tethered to the diaphragm, the diaphragm contour was defined by subtracting the lung contour at maximum expiration from the lung contour at maximum inspiration. The diaphragm contour was delineated using the average CT images and overridden to the density of muscle for optimization. The diaphragm override was then removed for final dose calculation and evaluation. Note that for all patients, the beam angles were selected to minimize the effect of diaphragm motion, and couch kicks were often utilized in cases where the target was located close to the diaphragm.

All plans were created, including PBS, in the treatment planning system (TPS) (RayStation Version 5.0.2.35). The dose calculation grid was set at 2 mm for all cases. The proton energies used ranged from 125 to 170 MeV, where the spot size varied from 3.8 to 4.8 mm when a range shifter of 75-mm water-equivalent thickness was used [30]. Volumetric repainting of either three or four times was used for the actual patient treatment plan, depending on the magnitude of tumor motion and the number of beam angles used. Both 3D and 4D optimizations used the same robustness settings: a range uncertainty of 4% was used in all plans with a uniform setup uncertainty of 5 mm in all directions.

The 4D robust optimization in the TPS can simultaneously optimize for all breathing phases in the 4D CT image set. In addition to accounting for all the robust scenarios in the average CT images similar to what is done in the 3D robust optimization, the 4D robust optimization examines all scenarios in the individual 4D CTs for the different breathing phases. All the 4D CT image sets can be included in the 4D robust optimization process, wherein all robust objectives will be optimized with all scenarios in all selected CT images. The resultant 4D optimized plan will, therefore, be robust against not only range and patient setup uncertainties but also breathing motions as captured by the 4D CT. To maximize plan robustness against the breathing motion, one would include all 4D CT breathing phases in the optimization process. However, with more breathing phases being included in the optimization, the computational load also increases proportionally. In practice, the number of CT images included in the optimization is limited by the available computational resources. In this study, we had chosen to include three breathing phases in addition to the average CT images for 4D optimization: maximum inspiration, maximum expiration and an intermediate breathing phase (termed “mid-phase” in Table 1) between them. An additional intermediate breathing phase (termed “mid-phase 2” in Table 1) that had not been included for 4D optimization was included for target and OAR dose statistics evaluation (see Table 1).

**Table 1:** Data summary Dosimetric comparison between 3D and 4D robust optimized plans. A total of seven lung patients have been studied using GTV D100%, CTV D99%, ITV D99% and ITV V70Gy as target coverage criteria. Lungs V5Gy and V20Gy are also examined. Dose statistics are summarized for the maximal inspiration, max expiration and two intermediate breathing phases (see text) and the average. Target coverage is seen to be more robust for 4D optimized plans, while OAR sparing showed little difference. Patient 6 and 7 had verification 4D CT after the treatment started and the plans were re-calculated on the VFCT. It is observed that the 4D optimized plans showed more robust target coverage.

			GTV D100%					CTV D99%					ITV D99%					ITV V70Gy					Lungs	
Patient	Technique	Rx	Max inspiration	Average scan	Mid- phase	Mid-phase 2	Max expiration	Max inspiration	Average scan	Mid-phase	Mid-phase 2	Max expiration	Max inspiration	Average scan	Mid-phase	Mid-phase 2	Max expiration	Max inspiration	Average scan	Mid-phase	Mid-phase 2	Max expiration	Lungs V5 average Scan	Lungs V20 average Scan
1	3D	70Gy	69.7 Gy	70.5 Gy	69.6 Gy	70.5 Gy	70.2 Gy	68.4 Gy	70.0 Gy	69.7 Gy	70.7 Gy	69.5 Gy	68.4 Gy	70.0 Gy	68.5 Gy	68.6 Gy	69.2 Gy	90.1%	99.0%	86.6%	91.6%	95.5%	10.2%	4.9%
	4D		70.3 Gy	70.5 Gy	70.6 Gy	70.8 Gy	71.3 Gy	70.1 Gy	70.0 Gy	70.6 Gy	70.9 Gy	70.0 Gy	70.4 Gy	70.0 Gy	70.2 Gy	70.0 Gy	70.6 Gy	98.5%	99.0%	99.5%	99.1%	99.9%	10.3%	5.1%
2	3D	70Gy	69.6 Gy	69.5 Gy	69.7 Gy	69.1 Gy	69.3 Gy	69.8 Gy	70.0 Gy	69.9 Gy	69.5 Gy	68.9 Gy	69.5 Gy	70.0 Gy	69.6 Gy	69.4 Gy	68.7 Gy	92.6%	99.0%	94.6%	94.0%	91.8%	14.9%	11.1%
	4D		70.8 Gy	70.2 Gy	70.8 Gy	70.0 Gy	70.0 Gy	70.6 Gy	70.0 Gy	70.5 Gy	70.0 Gy	70.2 Gy	70.2 Gy	70.0 Gy	70.2 Gy	70.0 Gy	70.0 Gy	99.6%	99.0%	99.5%	98.8%	99.0%	16.0%	12.0%
3	3D	70Gy	69.6 Gy	69.7 Gy	69.3 Gy	69.3 Gy	69.0 Gy	69.5 Gy	70.0 Gy	69.3 Gy	69.3 Gy	69.1 Gy	69.4 Gy	70.0 Gy	69.2 Gy	69.2 Gy	69.2 Gy	89.2%	99.0%	87.6%	86.9%	84.2%	12.0%	7.5%
	4D		70.4 Gy	70.3 Gy	70.3 Gy	70.2 Gy	70.0 Gy	70.0 Gy	70.0 Gy	69.9 Gy	70.1 Gy	70.1 Gy	70.0 Gy	70.0 Gy	70.0 Gy	70.0 Gy	70.0 Gy	98.9%	99.0%	99.1%	99.3%	99.2%	11.6%	7.1%
4	3D	70Gy	69.7 Gy	69.9 Gy	69.9 Gy	69.8 Gy	69.9 Gy	69.7 Gy	70.0 Gy	69.9 Gy	69.9 Gy	69.9 Gy	69.7 Gy	70.0 Gy	69.8 Gy	69.9 Gy	69.8 Gy	84.1%	99.0%	94.0%	96.3%	97.4%	10.1%	5.6%
	4D		70.1 Gy	70.2 Gy	70.3 Gy	70.5 Gy	70.3 Gy	70.1 Gy	70.0 Gy	70.2 Gy	70.4 Gy	69.9 Gy	70.0 Gy	70.0 Gy	70.1 Gy	70.1 Gy	69.9 Gy	98.7%	99.0%	99.2%	99.3%	98.7%	11.6%	6.7%
5	3D	70Gy	69.8 Gy	69.8 Gy	69.8 Gy	69.8 Gy	70.0 Gy	69.9 Gy	70.0 Gy	69.9 Gy	69.9 Gy	70.1 Gy	69.9 Gy	70.0 Gy	69.9 Gy	69.9 Gy	70.1 Gy	97.3%	99.0%	98.1%	98.3%	99.9%	6.7%	4.6%
	4D		70.1 Gy	70.2 Gy	70.2 Gy	70.1 Gy	70.1 Gy	69.9 Gy	70.0 Gy	70.1 Gy	70.1 Gy	70.2 Gy	69.9 Gy	70.0 Gy	70.0 Gy	70.0 Gy	70.1 Gy	98.8%	99.0%	99.2%	99.0%	99.2%	6.7%	4.6%
6	3D	70Gy	69.8 Gy	70.0 Gy	70.0 Gy	69.8 Gy	69.8 Gy	69.7 Gy	70.0 Gy	69.8 Gy	69.8 Gy	69.9 Gy	69.6 Gy	70.0 Gy	69.8 Gy	69.7 Gy	69.8 Gy	93.4%	99.0%	95.4%	92.7%	96.9%	10.7%	6.5%
	4D		70.8 Gy	70.9Gy	71.0 Gy	70.5 Gy	70.0 Gy	70.4 Gy	70.0 Gy	70.1 Gy	70.5 Gy	70.2 Gy	70.1 Gy	70.0 Gy	70.2 Gy	69.8 Gy	69.9 Gy	99.2%	99.0%	99.3%	98.5%	98.8%	10.7%	6.5%
7	3D	70Gy	69.7 Gy	70.0 Gy	70.1 Gy	70.0 Gy	70.2 Gy	69.9 Gy	70.0 Gy	70.1 Gy	70.2 Gy	70.3 Gy	69.8 Gy	70.0 Gy	69.9 Gy	69.9 Gy	69.6 Gy	98.0%	99.0%	98.7%	98.8%	98.2%	26.6%	15.4%
	4D		69.7 Gy	70.0 Gy	69.8 Gy	69.9 Gy	70.0 Gy	69.8 Gy	70.0 Gy	69.9 Gy	70.0 Gy	70.1 Gy	69.8 Gy	70.0 Gy	70.0 Gy	69.9 Gy	70.1 Gy	93.8%	99.0%	98.3%	98.0%	99.9%	29.0%	18.8%
6 VFCT	3D	70Gy	69.4 Gy	69.4 Gy	69.5 Gy	69.5 Gy	69.5 Gy	69.6 Gy	69.6 Gy	69.6 Gy	69.8 Gy	69.6 Gy	69.5 Gy	69.6 Gy	69.6 Gy	69.5 Gy	69.6 Gy	81.0%	85.7%	84.5%	84.7%	87.3%	11.8%	7.1%
	4D		70.1 Gy	70.2 Gy	69.8 Gy	70.5 Gy	69.7 Gy	70.1 Gy	68.6 Gy	69.8 Gy	70.1 Gy	69.9 Gy	68.8 Gy	68.6 Gy	69.2 Gy	68.5 Gy	69.5 Gy	95.3%	95.1%	99.3%	94.3%	96.4%	10.5%	6.0%
7 VFCT	3D	70Gy	68.8 Gy	69.4 Gy	69.3 Gy	69.4 Gy	68.6 Gy	67.8 Gy	68.6 Gy	68.8 Gy	68.8 Gy	67.6 Gy	67.4 Gy	68.6 Gy	68.1 Gy	68.4 Gy	67.0 Gy	47.6%	79.0%	69.5%	72.6%	75.8%	21.8%	12.3%
	4D		69.5 Gy	69.8 Gy	69.8 Gy	70.1 Gy	69.3 Gy	69.7 Gy	70.0 Gy	70.0 Gy	70.3 Gy	69.5 Gy	69.7 Gy	70.0 Gy	70.1Gy	70.3 Gy	69.6 Gy	96.1%	99.2%	99.4%	100.0%	94.6%	23.6%	14.7%

All plans were normalized such that the ITV D99% on the average CT was equal to the prescription dose of 70 Gy(RBE). Additional evaluations were performed using verification CT scans on two patients (patients 6 and 7) at two to three weeks after the first treatment fraction. Both 3D and 4D optimized plans were re-calculated on the verification CT, and the various target coverage and OAR criteria were examined. The purpose of this verification CT is to evaluate the robustness of the plan against inter-fractional variations. Using rigid registration, the locations of the tumor were aligned between the verification CT and the original planning CT. This rigid registration mimics the daily image-guided setup of the patient.

## Results

All plans were evaluated by target coverages, i.e., GTV at D100%, CTV at D99%, ITV at D99% and ITV at V70Gy, for individual breathing phases and on the average CT. Table 1 summarizes the results of this evaluation for three individual breathing phases: maximal inspiration, maximal expiration and an intermediate breathing phase included in the 4D optimization (i.e., the “mid-phase”), as well as another intermediate breathing phase not included in the 4D optimization (i.e., the “mid-phase 2”). Sparing of OAR was evaluated on the average CT using the lungs criteria – V5Gy and V20Gy.

**Figure 2:**
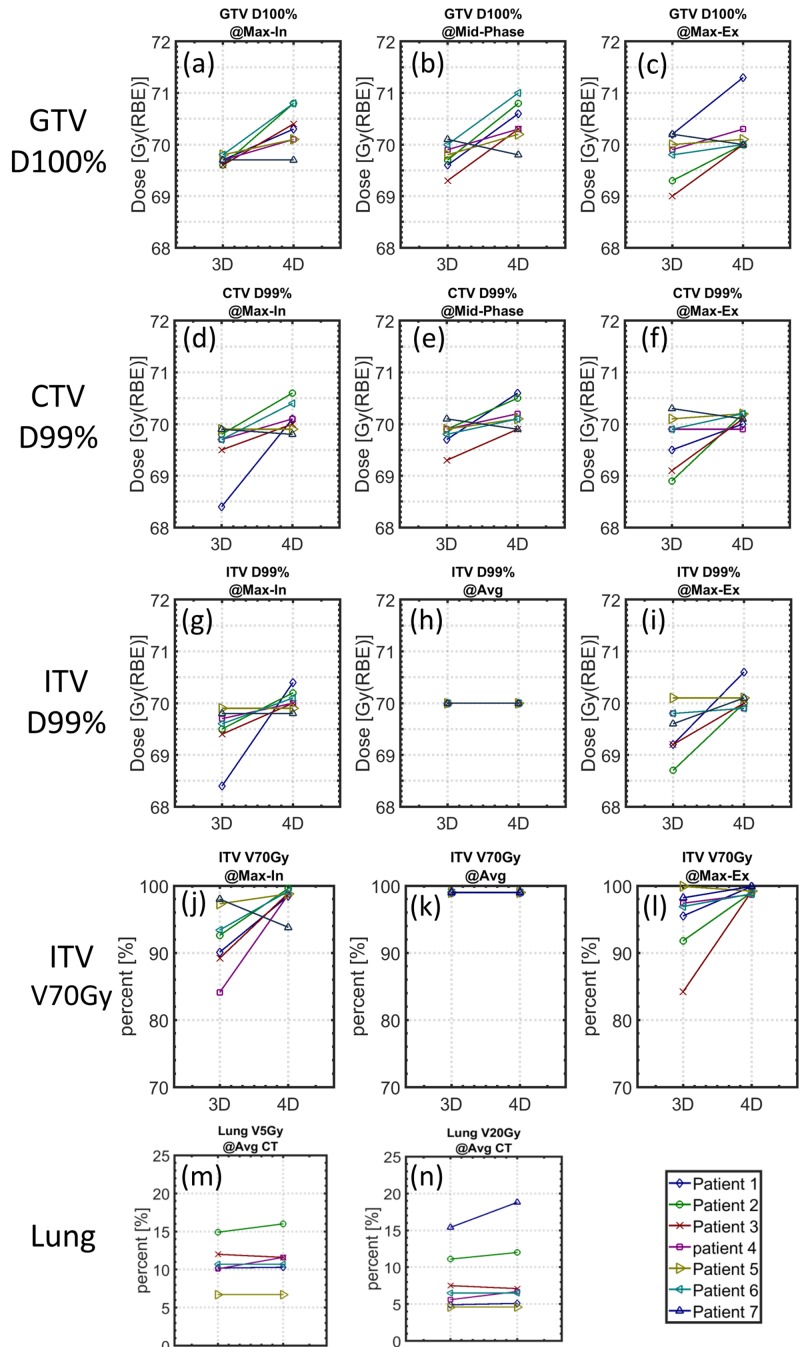
Dose-volume histogram comparison Dosimetric comparison between 3D and 4D robust optimized plans. All plans are normalized to ITV D99% at 70 Gy(RBE) on the average CT, as seen in (h) and (k). The 4D optimized plans generally show better target coverage with negligible lung difference. 3D: three-dimensional, 4D: four-dimensional, ITV: internal target volume, CT: computed tomography

In six of the seven patients examined, evaluation in all the individual breathing phases showed that the GTV coverage at D100% of the 4D optimized plans was slightly greater than or equal to that of its 3D counterpart. A similar trend was observed while evaluating the CTV coverage, i.e., the CTV coverage at D99% of the 4D optimized plans was typically higher than that of the 3D optimized plans in all the individual breathing phases. This observation was consistent with the fact that the 4D plans were specifically optimized on the individual breathing phase CTs in addition to the average CT, while the 3D plans were optimized on the average CT only. Table 1 summarizes the GTV and CTV coverages of each patient in various breathing phases. Figure 2 shows a paired comparison of these target coverages.

**Figure 3:**
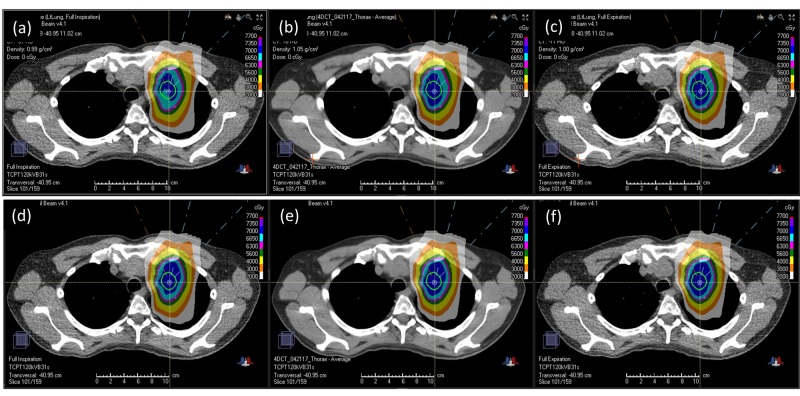
Dose distribution comparison Comparison between 3D robust optimization (a, b, c) and 4D robust optimization (d, e, f) on the planning CT. The 3D plan is optimized on the average CT (b), and calculated on the maximal inspiration phase (a) and the maximal expiration phase (c). The 4D plan is optimized simultaneously on the maximal inspiration phase (d), the average CT (e), and the maximal expiration phase (f). Note that the 4D plan is optimized simultaneously on the average CT, the maximal inspiration phase, the maximal expiration phase, and an intermediate breathing phase (not shown). The contour shown in all panels is the ITV. The 4D optimized plan shows more consistent ITV coverage. Lung sparing between these two plans are comparable (details in text). 3D: three-dimensional, 4D: four-dimensional, CT: computed tomography, ITV: internal target volume

The difference between the 4D and 3D optimized plans in terms of target coverage robustness was most pronounced when evaluated using ITV V70Gy. While all plans were normalized such that the ITV V70Gy coverage equalled 99% on the average CT, the same V70Gy measure exhibited observably varied coverage when the plans were evaluated for the maximal inspiration and expiration phases. As seen in Table 1, the ITV V70Gy coverage in the maximal inspiration phase varied between 93.8% and 99.6% for the 4D optimized plans, while for the 3D optimized plans, this variation exacerbated from 84.1% to 98.0%. The same phenomenon was also observed in the maximal expiration phase, where 4D optimized plans maintained a lower bound of 98.7% for V70Gy as opposed to only 84.2% for the 3D optimized plans. Again, for the same reason mentioned earlier for GTV and CTV, some degradation in the ITV coverage for the 3D plan was expected, as 3D plans are not specifically optimized for individual breathing phases. Figure 3 illustrates this difference in ITV V70Gy coverages for the 4D and 3D optimized plans. It is observed that 4D optimized plans consistently maintained ITV coverage throughout the breathing cycle, while ITV coverage in 3D optimized plans can potentially deteriorate significantly. 

This study did not show a significant difference in OAR sparing between the 3D and 4D optimized plans, i.e., for all seven cases studied herein, the difference between the 4D and 3D optimization plans in terms of the lungs criteria, V5Gy and V20Gy, was relatively small (<3.4%). Nevertheless, the lung V5Gy and V20Gy percentages were in general slightly higher for the 4D optimized plans in four of the seven cases (patient 1, 2, 4 and 7). Note that 4D plans observed a slightly higher lung dose, which is in line with its better target coverage. The results here show that compared with the 3D optimization, 4D optimization can achieve better target coverage while maintaining a negligible difference in the OAR dose level.

**Figure 4:**
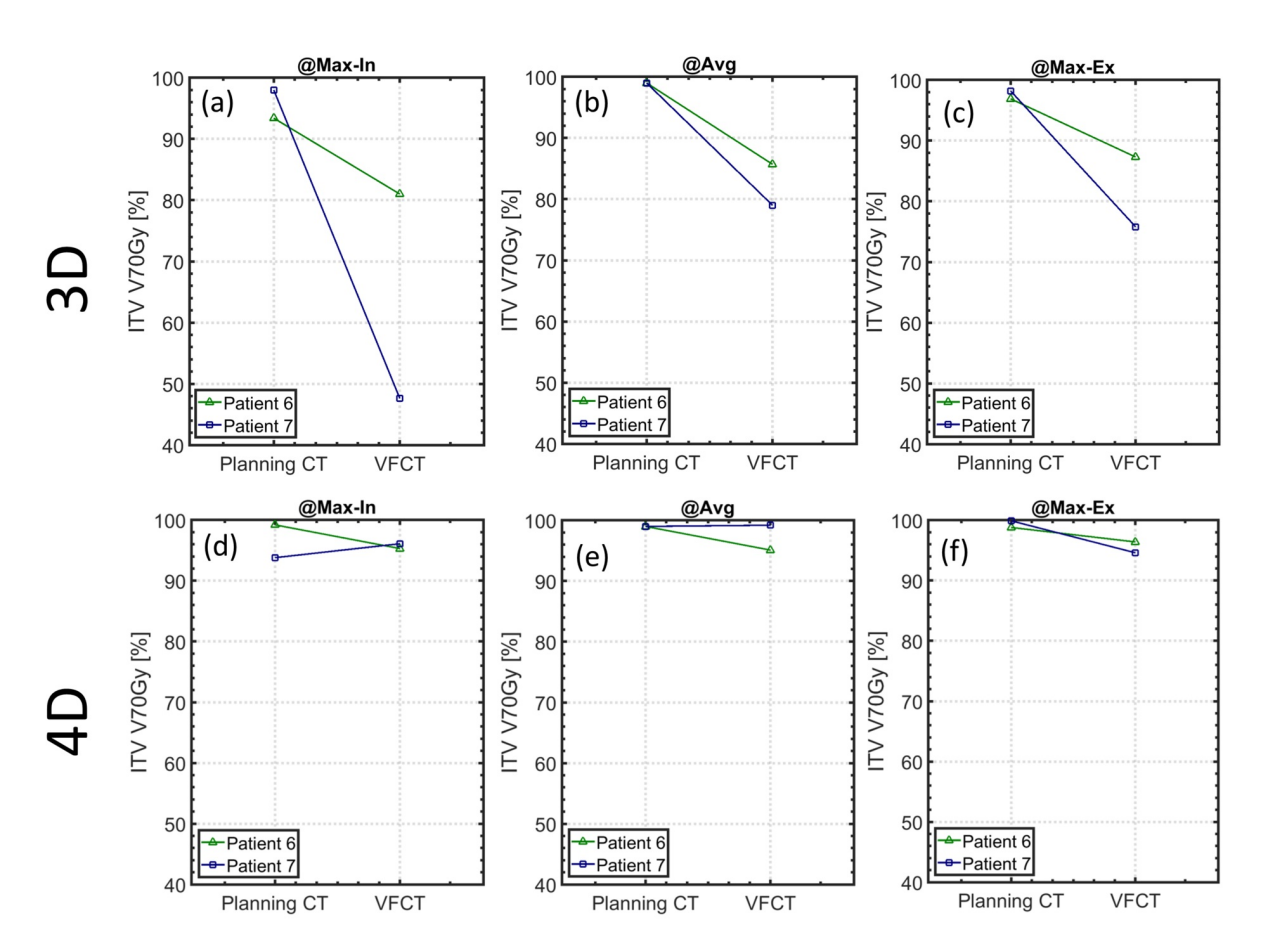
Verification computed tomography: dose-volume histogram Evaluation of 3D vs. 4D plan robustness using VFCT scans. The internal target volume (ITV) V70Gy is used as the criterion. The 3D optimized plans are shown in the top row (a, b, c), and the 4D optimized plans in the bottom row (d, e, f). The 3D plans were robustly optimized on the average CT and evaluated on the maximum inspiration and maximum expiration phases of the planning CTs. The 3D plan’s target coverage is indeed ensured on the average planning CT (b), but is not guaranteed on the individual breathing phases of the planning CTs (a) and (c). Specifically, for patient 6, the 3D plan’s target coverage is less than 80% on the maximum inspiration and maximum expiration phases of the planning CT, while for patient 7, the 3D plan has >98% coverage. More importantly, the 3D plan’s target coverage, when evaluated on the VFCT, was consistently lower than 80% for both patient 6 and 7, even on the average VFCT. On the contrary, 4D optimized plans showed consistent target coverage on all phases of the planning CT as well as the VFCT (d, e, f). While good target coverage in all phases of the planning CT is indeed expected of the 4D optimized plans since it is specifically optimized on those specific CTs, these 4D optimized plans also demonstrated better target coverage across all phases in the VFCT. This suggested that 4D optimized plans are more robust against inter-fractional motions than 3D optimized plans. 3D: three-dimensional, 4D: four-dimensional, VFCT: verification computed tomography, CT: computer tomography

**Figure 5:**
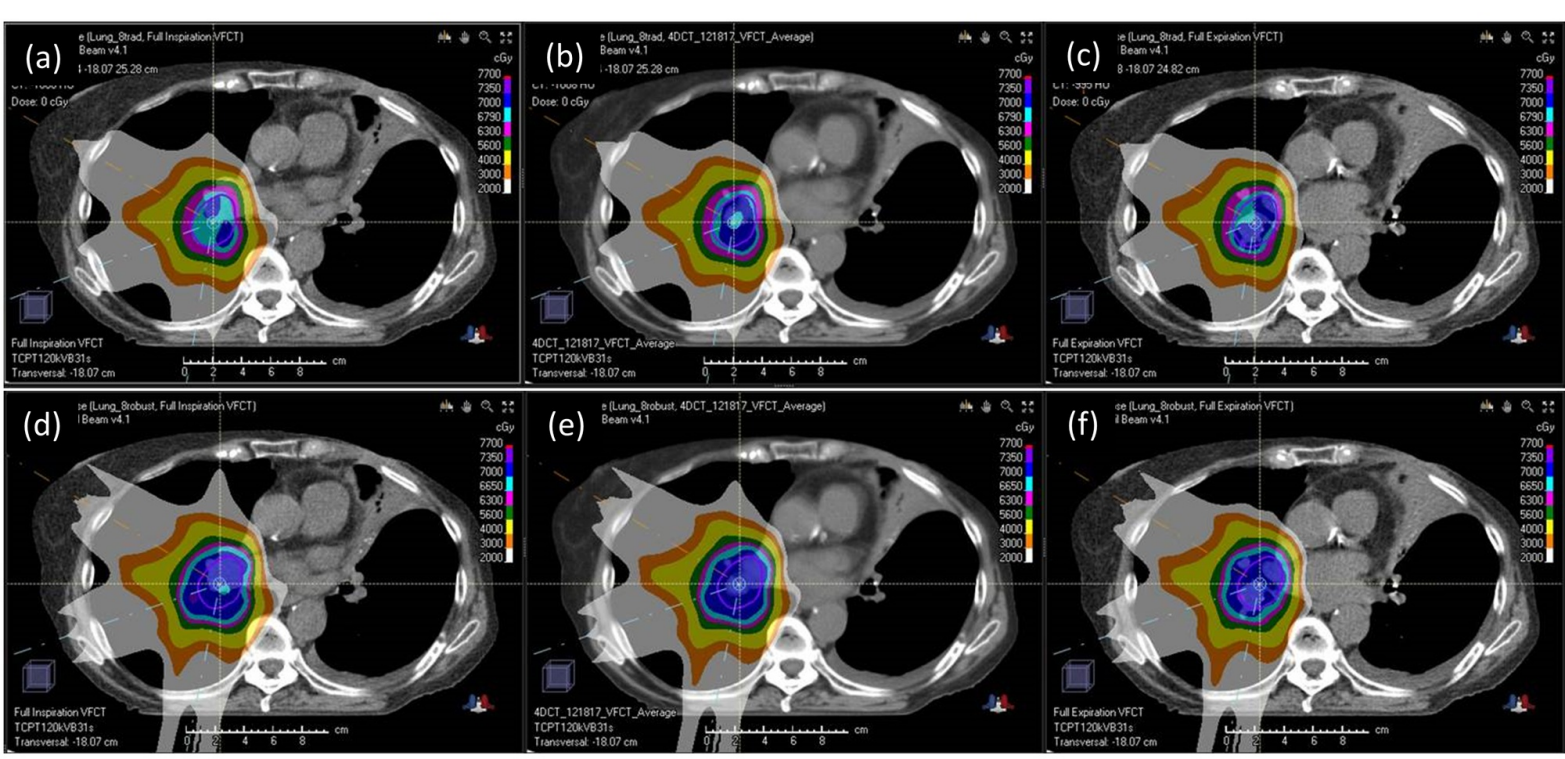
Verification computed tomography: dose distribution Comparison between the 3D optimized plan (a, b, c) and 4D optimized plan (d, e, f), when calculated on the VFCT. The 3D optimized plan is calculated on the VFCT's maximal inspiration phase (a), average (b), and the maximal expiration phase (c). The 4D optimized plan is calculated on the same VFCT scans, i.e., maximal inspiration (d), average (e), and maximal expiration (f). Note that the 3D plan’s target coverage deteriorated on all the VFCT scans, while that of the 4D optimized plan only showed minor degradation. This shows that 4D optimized plans are indeed more robust against inter-fractional variations. This is most likely due to the fact that 4D optimized plans have more stringent robustness criteria when being optimized (details in text). 3D: three-dimensional, 4D; four-dimensional, VFCT: verification computed tomography

On the verification CT (VFCT), it was generally observed that the 4D optimized plans maintained a better target coverage than the 3D optimized plans for all the breathing phases as well as the average scan. Both 4D and 3D optimized plans showed similar OAR sparing when evaluated on the VFCT, i.e., differences in lungs V5Gy and V20Gy are within 2.4% (Table 1, bottom two rows). Figure 4 presents a comparison of ITV V70Gy between the 3D and 4D optimized plans when calculated on the VFCT’s maximum inspiration phase (a, d), average (b, e), and maximum expiration phase (c, f). As seen in Figure 4 (a, b, c), the ITV V70Gy of the 3D optimized plans is significantly lower for both patient 6 and 7 on all VFCTs when compared with that of the 4D optimized plans. Note that patient 7’s 3D optimized plan, despite having a greater than 98% ITV V70Gy coverage on the planning CT’s various individual phases and the average scan, still ended up with less than 80% coverage when calculated on the corresponding phases of the VFCT. Its 4D optimized counterpart, on the other hand, had greater than 93.8% ITV V70Gy coverage on all planning and VFCT images. For patient 6, the 3D optimized plan, which was normalized to 99% on the planning CT’s average scan, already showed <93% coverage on one of the planning CT phases. Not surprisingly, this 3D plan's ITV V70Gy coverage on the VFCTs was also consistently less than 90%. For comparison, patient 6’s 4D optimized plan maintained an ITV V70Gy coverage greater than 98.5% for all the breathing phases in the planning CT and greater than 94.3% in the VFCTs. Specifically, the ITV V70Gy coverage of patient 6’s 4D optimized plan only reduced slightly from 99.0% to 95.1% on the average scans between planning CT and VFCT, while that of the 3D optimized plan deteriorated significantly from 99.0% to 85.7%. For patient 7, the ITV V70Gy coverage of the 3D plan dropped to 79.0% on the average VFCT scan, while its 4D plan maintained the ITV V70Gy coverage at 99.2%. The 4D optimized plans, therefore, maintained a more robust ITV coverage against inter-fractional variations, as evidenced by the higher ITV V70Gy coverage when evaluated on the VFCTs. Figure 5 shows an example of the axial dose distributions of patient 7’s 3D (a, b, c) and 4D (d, e, f) plans, calculated on various phases and the average of VFCTs.

## Discussion

The 4D robust optimization method, like its 3D counterpart, generates only one treatment plan for the patients, although it is obtained by simultaneously optimizing on multiple CT image sets. One may argue that the ITV coverage need not be maintained throughout the entire breathing cycle during planning; thus, the advantage of the 4D optimized plans in the maximal inspiration and maximal expiration phases may not be clinically significant. However, given that this advantage is indeed observed in the VFCT, ITV coverage in the individual breathing phases can still serve as a viable indicator for the robustness of a PBS plan against tumor motion. Other motion mitigation strategies, such as multi-gating and active breathing control, may still be required in combination with 4D robust optimization to better mitigate tumor motion for the proton PBS treatment of lung tumors.

This study demonstrated the clinical use of 4D robust optimization, which is capable of taking into account simultaneously all the breathing phases of the 4D CT during optimization. Compared with the optimizations that utilize only one average CT scan, this 4D robust optimization technique is shown to be more robust in maintaining the target coverage in all individual breathing phases while maintaining similar levels of OAR sparing. Most importantly, it is observed that the 4D optimized plan maintained a better ITV coverage when evaluated on the verification CT. As a result, we conclude that 4D optimizations can potentially provide a more robust treatment against inter-fractional variations. This advantage of 4D robust optimization in target coverage over 3D robust optimization is most likely due to the fact that the CTV contours of the individual phases were all specifically optimized by incorporating target objectives during plan optimization. The optimization objectives that spare the lungs, on the other hand, were calculated on the average CT scan only, which likely resulted in the nebulous lung sparing effectiveness observed here between the 4D and the 3D treatment plans.

## Conclusions

Radiation treatment planning for lung cancer has always proven to be difficult due to tumor motion during the respiratory cycle. This study examined the clinical effectiveness of the newly available 4D robust optimization functionality in a commercially available TPS. By simultaneously optimizing on individual breathing phases, the advantage of 4D robust optimization over 3D robust optimization was demonstrated by not only the better target coverage in individual breathing phases during planning, as is expected of 4D optimization, but also the more robust target coverages on the verification CT, which indicated the robustness of 4D optimized plans against inter-fractional variations.

## References

[1] Siegel RL, Miller KD, Jemal A (2017). Cancer statistics, 2017. CA Cancer J Clin.

[2] Chang JY, Zhang W, Komaki R (2017). Long-term outcome of phase I/II prospective study of dose-escalated proton therapy for early-stage non-small cell lung cancer. Radiother Oncol.

[3] Wang XS, Shi Q, Williams LA (2016). Prospective study of patient-reported symptom burden in patients with non-small-cell lung cancer undergoing proton or photon chemoradiation therapy. J Pain Symptom Manage.

[4] Chang JY, Jabbour SK, De Ruysscher D (2016). Subcommittee IPTC-oGT: consensus statement on proton therapy in early-stage and locally advanced non-small cell lung cancer. Int J Radiat Oncol Biol Phys.

[5] Grassberger C, Dowdell S, Sharp G, Paganetti H (2015). Motion mitigation for lung cancer patients treated with active scanning proton therapy. Med Phys.

[6] Stuschke M, Kaiser A, Pöttgen C, Lübcke W, Farr J (2012). Potentials of robust intensity modulated scanning proton plans for locally advanced lung cancer in comparison to intensity modulated photon plans. Radiother Oncol.

[7] Li Y, Kardar L, Li X (2014). On the interplay effects with proton scanning beams in stage III lung cancer. Med Phys.

[8] Kardar L, Li Y, Li X (2014). Evaluation and mitigation of the interplay effects of intensity modulated proton therapy for lung cancer in a clinical setting. Pract Radiat Oncol.

[9] Grassberger C, Daartz J, Dowdell S, Ruggieri T, Sharp G, Paganetti H (2014). Quantification of proton dose calculation accuracy in the lung. Int J Radiat Oncol Biol Phys.

[10] Dowdell S, Grassberger C, Sharp GC, Paganetti H (2013). Interplay effects in proton scanning for lung: a 4D Monte Carlo study assessing the impact of tumor and beam delivery parameters. Phys Med Biol.

[11] Kraus KM, Heath E, Oelfke U (2011). Dosimetric consequences of tumour motion due to respiration for a scanned proton beam. Phys Med Biol.

[12] Paganetti H, Jiang H, Trofimov A (2005). 4D Monte Carlo simulation of proton beam scanning: modelling of variations in time and space to study the interplay between scanning pattern and time-dependent patient geometry. Phys Med Biol.

[13] Paganetti H, Jiang H, Adams JA, Chen GT, Rietzel E (2004). Monte Carlo simulations with time-dependent geometries to investigate effects of organ motion with high temporal resolution. Int J Radiat Oncol Biol Phys.

[14] Bortfeld T, Jokivarsi K, Goitein M, Kung J, Jiang SB (2002). Effects of intra-fraction motion on IMRT dose delivery: statistical analysis and simulation. Phys Med Biol.

[15] Liu W, Schild SE, Chang JY (2016). Exploratory study of 4D versus 3D robust optimization in intensity modulated proton therapy for lung cancer. Int J Radiat Oncol Biol Phys.

[16] Liu W, Liao Z, Schild SE (2015). Impact of respiratory motion on worst-case scenario optimized intensity modulated proton therapy for lung cancers. Pract Radiat Oncol.

[17] Mori S, Wolfgang J, Lu HM, Schneider R, Choi NC, Chen GT (2008). Quantitative assessment of range fluctuations in charged particle lung irradiation. Int J Radiat Oncol Biol Phys.

[18] Seco J, Robertson D, Trofimov A, Paganetti H (2009). Breathing interplay effects during proton beam scanning: simulation and statistical analysis. Phys Med Biol.

[19] Lin L, Souris K, Kang M, Glick A, Lin H, Huang S, Stützer K, Janssens G (2017). Evaluation of motion mitigation using abdominal compression in the clinical implementation of pencil beam scanning proton therapy of liver tumors. Med Phys.

[20] Graeff C (2017). Robustness of 4D-optimized scanned carbon ion beam therapy against interfractional changes in lung cancer. Radiother Oncol.

[21] Brevet R, Richter D, Graeff C, Durante M, Bert C (2015). Treatment parameters optimization to compensate for interfractional anatomy variability and intrafractional tumor motion. Front Oncol.

[22] Eley JG, Newhauser WD, Lüchtenborg R, Graeff C, Bert C (2014). 4D optimization of scanned ion beam tracking therapy for moving tumors. Phys Med Biol.

[23] Richter D, Schwarzkopf A, Trautmann J, Krämer M, Durante M, Jäkel O, Bert C (2013). Upgrade and benchmarking of a 4D treatment planning system for scanned ion beam therapy. Med Phys.

[24] Gorgisyan J, Munck af Rosenschold P, Perrin R (2018). Feasibility of pencil beam scanned intensity modulated proton therapy in breath-hold for locally advanced non-small cell lung cancer. Int J Radiat Oncol Biol Phys.

[25] Wang N, Patyal B, Ghebremedhin A, Bush D (2013). Evaluation and comparison of New 4DCT based strategies for proton treatment planning for lung tumors. Radiat Oncol.

[26] Kang Y, Zhang X, Chang JY (2007). 4D Proton treatment planning strategy for mobile lung tumors. Int J Radiat Oncol Biol Phys.

[27] Muirhead R, McNee SG, Featherstone C, Moore K, Muscat S (2008). Use of maximum intensity projections (MIPs) for target outlining in 4DCT radiotherapy planning. J Thorac Oncol.

[28] Rietzel E, Liu AK, Chen GT, Choi NC (2008). Maximum-intensity volumes for fast contouring of lung tumors including respiratory motion in 4DCT planning. Int J Radiat Oncol Biol Phys.

[29] Underberg RW, Lagerwaard FJ, Slotman BJ, Cuijpers JP, Senan S (2005). Use of maximum intensity projections (MIP) for target volume generation in 4DCT scans for lung cancer. Int J Radiat Oncol Biol Phys.

[30] Wang P, Tang S, Taylor PA (2018). Clinical examination of proton pencil beam scanning on a moving anthropomorphic lung phantom. Med Dosi.

